# WNK signalling pathways in blood pressure regulation

**DOI:** 10.1007/s00018-016-2402-z

**Published:** 2016-11-04

**Authors:** Meena Murthy, Thimo Kurz, Kevin M. O’Shaughnessy

**Affiliations:** 1 Division of Experimental Medicine and Immunotherapeutics, Department of Medicine, University of Cambridge, Cambridge, CB2 2QQ UK; 2 Institute of Molecular Cell and Systems Biology, University of Glasgow, Davidson Building, Glasgow, G12 8QQ Scotland, UK

**Keywords:** WNK kinases, SPAK/OSR1 phosphorylation, NCC, Ubiquitin-E3 ligase complex, Proteasome, Hyperkalemia, Hypertension

## Abstract

Hypertension (high blood pressure) is a major public health problem affecting more than a billion people worldwide with complications, including stroke, heart failure and kidney failure. The regulation of blood pressure is multifactorial reflecting genetic susceptibility, in utero environment and external factors such as obesity and salt intake. In keeping with Arthur Guyton’s hypothesis, the kidney plays a key role in blood pressure control and data from clinical studies; physiology and genetics have shown that hypertension is driven a failure of the kidney to excrete excess salt at normal levels of blood pressure. There is a number of rare Mendelian blood pressure syndromes, which have shed light on the molecular mechanisms involved in dysregulated ion transport in the distal kidney. One in particular is Familial hyperkalemic hypertension (FHHt), an autosomal dominant monogenic form of hypertension characterised by high blood pressure, hyperkalemia, hyperchloremic metabolic acidosis, and hypercalciuria. The clinical signs of FHHt are treated by low doses of thiazide diuretic, and it mirrors Gitelman syndrome which features the inverse phenotype of hypotension, hypokalemic metabolic alkalosis, and hypocalciuria. Gitelman syndrome is caused by loss of function mutations in the thiazide-sensitive Na/Cl cotransporter (NCC); however, FHHt patients do not have mutations in the SCL12A3 locus encoding NCC. Instead, mutations have been identified in genes that have revealed a key signalling pathway that regulates NCC and several other key transporters and ion channels in the kidney that are critical for BP regulation. This is the WNK kinase signalling pathway that is the subject of this review.

## Introduction

**Fig. 1:**
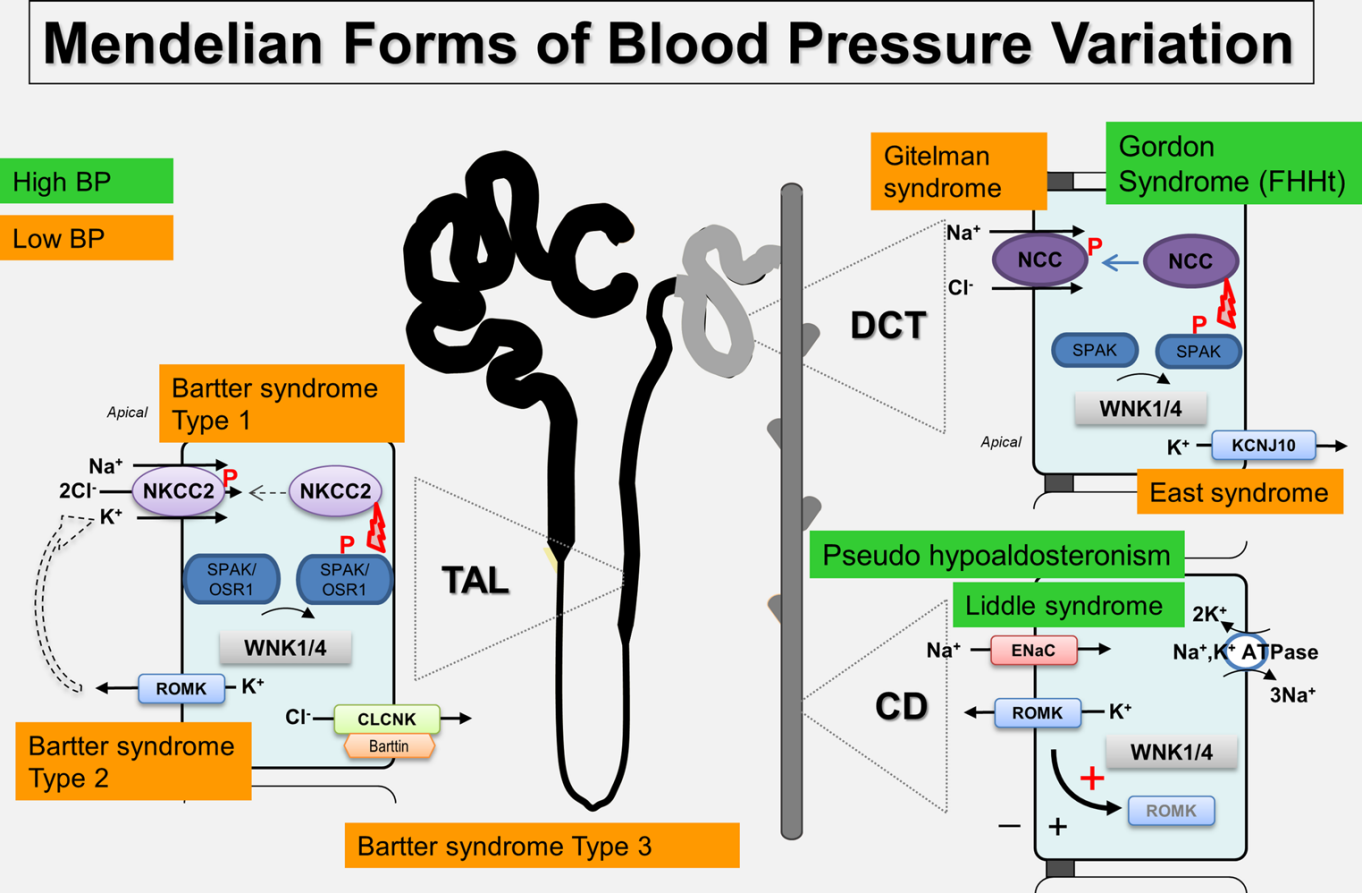
Diagram of the human nephron showing the locations where the main Mendelian syndromes affecting BP operate and the molecular mechanisms involved. The Na^+^, K^+^-ATPase is expressed along the nephron but due to space limitations is only shown in the CD. Abbreviations of nephron segments: *CD* collecting duct, *DCT* distal convoluted tubule, *TAL* thick ascending limb

Hypertension (high blood pressure) is a major public health problem affecting more than a billion people worldwide with complications, including stroke, heart failure and kidney failure [1]. The regulation of blood pressure (BP) is multifactorial reflecting genetic susceptibility, in utero environment and external factors such as obesity and salt intake. In keeping with Arthur Guyton’s hypothesis, the kidney plays a key role in blood pressure control [2] and data from clinical studies; physiology and genetics have shown that hypertension is driven by a failure of the kidney to excrete excess salt at normal levels of blood pressure. There is a number of rare Mendelian blood pressure syndromes (Fig. 1), which have shed light on the molecular mechanisms involved in dysregulated ion transport in the distal kidney. One in particular is Familial hyperkalemic hypertension (FHHt), a monogenic form of hypertension characterised by high blood pressure, hyperkalemia, hyperchloremic metabolic acidosis, and hypercalciuria [3]. The clinical signs of FHHt are treated by low doses of thiazide diuretics [4], and it mirrors to an extent Gitelman syndrome [5] which features the inverse phenotype of hypotension, hypokalemic metabolic alkalosis, hypomagnesemia, and hypocalciuria. However, it is worth emphasising that FHHt patients are typically normomagnesemic and Gitelman patients are usually normotensive. Gitelman syndrome is caused by loss of function mutations in the thiazide-sensitive Na/Cl cotransporter (NCC), but FHHt patients do not have mutations in the *SCL12A3* locus encoding NCC. Instead, mutations have been identified in genes that have revealed a key signalling pathway that regulates NCC and several other key transporters and ion channels in the kidney that are critical for BP regulation. This is the WNK kinase signalling pathway that is the subject of this review.

## WNK kinases

**Fig. 2:**
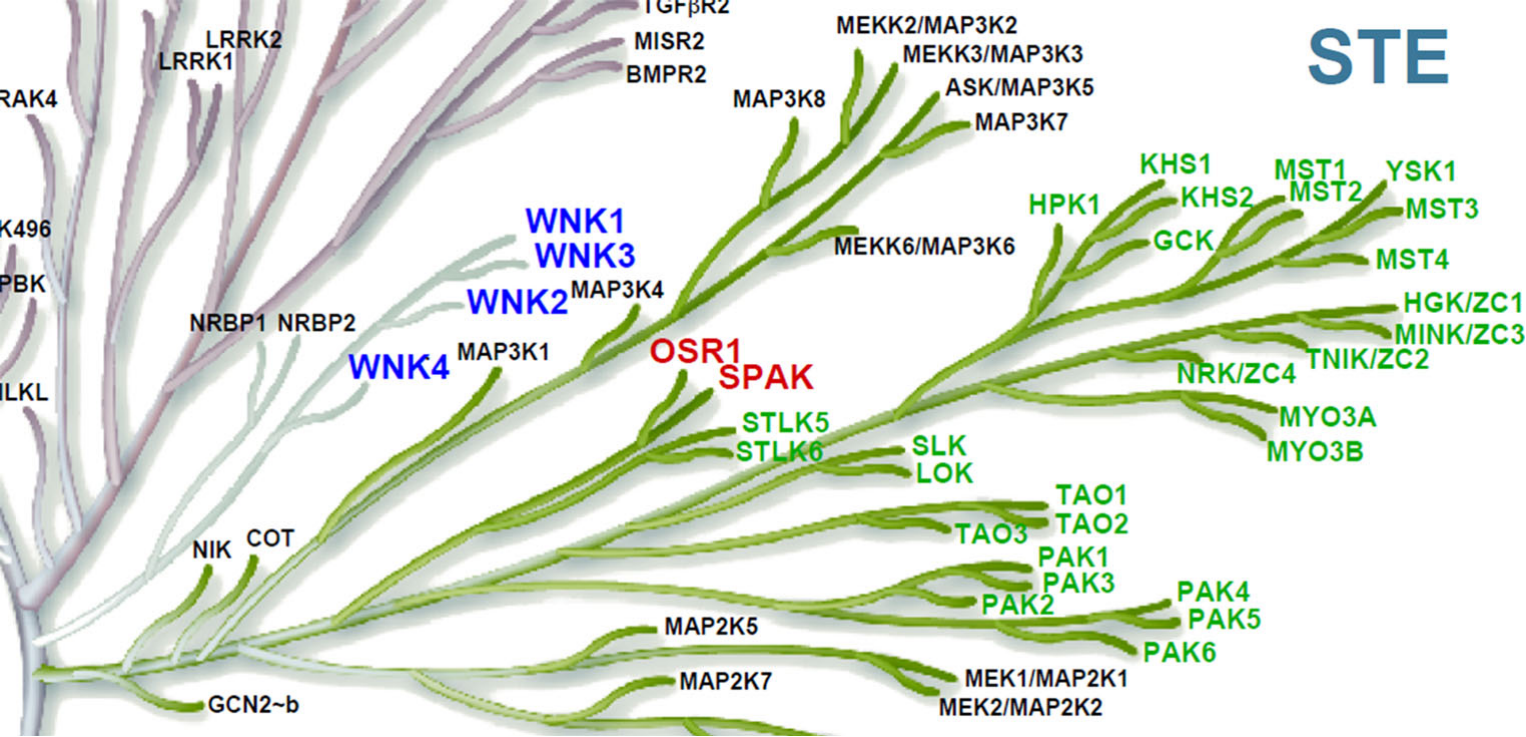
Zoomed section of the human kinome to show the close evolutionary proximity of WNKs and OSR1/SAPK From reference [6] with permission

The WNK kinases are a family of four evolutionarily conserved serine–threonine kinases (WNK1, WNK2, WNK3 and WNK4) that share >85% homology over their kinase domains and form a distinct branch of the phylogenetic tree of the human kinome (Fig. 2) [6]. However, unlike other kinases they use a catalytic Lys residue downstream from the usual site deep in the kinase core (kinase subdomain I). Hence, the term WNK (With No Lys (K)) referring to the absence of the usual N-terminal canonical kinase Lys residue for docking ATP and phosphoryl transfer (e.g. Lys^72^ in Protein Kinase A). This shift to a more superficial and distal glycine-rich loop for their canonical Lys has allowed WNKs to adapt their function and roles by acquiring an important sensitivity to chloride [7] (see “Intracellular Cl^−^ modulates activity of WNK kinases”). Overlap of the ‘chloride sensor’ in WNKs with the proximal canonical Lys residue explains the use of a distal Lys residue in the WNKs for their kinase activity (e.g. Lys^233^ in WNK1). This unique feature has lead to changes to WNK tertiary structure recently exploited in the development of a highly WNK-selective inhibitor (see “WNK/SPAK/OSR as a druggable signalling pathway”).

Another key property of the WNK kinases directly related to their ‘chloride sensor’ behaviour is inactive and active forms; with phosphorylation stabilising the active state [7]. Chloride anions inhibit this autophosphorylation, which explains how WNK kinase activity can respond to changes in intracellular chloride concentration [Cl^−^] and tonicity [8, 9]. This low Cl^−^ activation occurs rapidly (in <0.5 min) and involves phosphorylation of Ser^382^ in the T-loop of WNK1, which is conserved across all the WNKs [10]. The discovery of the ‘chloride sensor’ is recent, but follows long-standing speculation about the existence of a chloride-sensing regulatory kinase to explain the behaviour of Na^+^ and K^+^ cation cotransporters (NKCCs) in determining [Cl^−^] [11]. The need for this level of control reflects the importance of intracellular chloride in regulating cell volume itself, neuronal function and cell growth [12]. Recent crystallographic data has identified an LGL motif dubbed the ‘chloride sensor’ in WNK1 that confers chloride sensitivity by blocking the autophosphorylation of the T-loop [7]. This discovery of the chloride-sensing capacity of the WNKs has confirmed them as the ‘missing-link’ kinase in chloride regulation. It seems likely that WNK1 played a pivotal evolutionary role in controlling cell volume in single cells, although the only unicellular organism with a WNK1 orthologue identified so far is the dimorphic fungus *Penicillium marneffei* (GenBank: KFX50394.1). The development of closed cardiovascular systems in larger complex metazoan organisms may have necessitated the refinement of its volume regulatory function with gene duplication deriving later WNKs (WNK2–4). What is clear is that the WNKs now have a very diverse biology and a central role in the control of blood pressure.

## Inherited hypertension and WNK signalling

Four different genes have been implicated in FHHt (Table 1; Fig. 1) and two of them encode for WNK kinases: *WNK1* and *WNK4*. The *WNK1* sequence was first identified in 2000 from a rat brain cDNA library [13], but its function was unclear until intronic mutations were identified in the *WNK1* gene in human pedigrees segregating the FHHt phenotype. These mutations were associated with elevated levels of *WNK1* messenger RNA in peripheral monocytes from the affected patients and further missense mutations were identified in the orthologous *WNK4* gene of other unrelated pedigrees [14]. The other two FHHt genes, *CUL3* (Cullin3) and *KLHL3* (Kelch-like 3), which were discovered through a whole exome sequencing strategy, regulate WNK kinase levels in the cell. Together Cullin3 and KLHL3 form a Cullin-RING type E3 ubiquitin ligase complex that targets WNK kinases for ubiquitination to promote their proteasomal degradation. Cullin3 is a scaffold protein that coordinates the enzymatic and substrate binding activities of the ubiquitin E3 ligase. KLHL3 is the substrate receptor protein that recruits WNKs to the E3 ligase complex to present them for ubiquitination. Once ubiquitinated, WNKs are degraded by the 26S proteasome. Mutations in *CUL3* cause a dominantly inherited severe form of FHHt [15, 16]. It has been suggested that these mutations may increase ubiquitination and degradation of KLHL3, preventing WNK recruitment to the ligase complex, and thus indirectly abolishing WNK4 degradation [17, 18]. However, other work has suggested that the mutations affect the molecular flexibility of Cullin3 and lead to its auto-degradation by auto-ubiquitination, without affecting KLHL3 levels [16] (“CUL3-KLHL3 as upstream regulators of WNK kinases”). In contrast to *CUL3* mutations, mutations in *KLHL3* can result in either a dominant or recessive form of FHHt [18]. KLHL3 interacts directly with the CUL3 substrate (WNK in this case) and some recessive KLHL3 mutations directly inhibit this interaction. This prevents WNK ubiquitination and degradation further supporting the notion that elevated WNK levels lead to hypertension [17].

**Table 1:** FHHt mutations and their effects on the affected genes

Gene	Effect	Result	Effect on the encoded protein	References
*WNK1*	Deletion of intron I	↑ WNK1 expression	↑ L-WNK1 expression	[14, 25]
*WNK4*	Missense mutation in the acidic motif	↑ WNK4 expression due to disruption in the KLHL3 recognition site	↑ WNK4	[14, 15, 17, 81]
*WNK4*	R1185C mutation in the C-terminal domain	Disrupts a regulatory mechanism involving calmodulin binding and SGK1 phosphorylation sites	Unknown	[26, 27]
*KLHL3*	Missense mutations in the BTB or BACK domain	Disruption of the CUL3–KLHL3 interaction	↑ WNK1 ↑ WNK4 ↑ WNK3	[15, 17, 18, 81, 82]
	Missense mutations in the Kelch propeller blades	Disruption of the substrate (WNK) binding	↑ WNK1 ↑ WNK4 ↑ WNK3	
*CUL3*	Exon 9 deletion	Increased KLHL3 ubiquitination and degradation	↓KLHL3 ↑ WNK1 ↑ WNK4 ↑ WNK3	[15, 17, 86]
		Altered CUL3 flexibility leading to CUL3 auto-degradation and prevention of WNK ubiquitination		[16]

↑ indicates increase, ↓ indicates decrease

The association of mutations in the WNK kinases with FHHt suggested that an entirely novel pathway existed, connecting WNK kinases with renal electrolyte homeostasis and blood pressure. The last 15 years has been spent unravelling the molecular basis and complexity of this pathway. A crucial feature is the ability of WNK signalling to coordinate two competitive aldosterone-controlled processes: NaCl reabsorption (from the urine to the blood) and K^+^ secretion (from the blood to the urine) in the distal nephron to regulate blood pressure and maintain electrolyte homeostasis [19]. Work from groups in Yale and Oregon showed that this was achieved by WNKs regulating the phosphorylation and activities of cation-chloride cotransporters (CCCs), including NCC (in the DCT, distal convoluted tubule), KCC4 and NKCC2 (in the TAL, thick ascending limb) [20–22] and the ROMK channel [23], and the epithelial Na^+^ channel ENaC in the distal tubule and collecting duct [24]. The FHHt mutations reported in *WNK1* [14, 25] are large deletions of the first intron that result in an increased expression of WNK1 message, whereas those in *WNK4* are missense mutations that are clustered in the highly conserved acidic motif [26]. To date, only one of the *WNK4* mutations has been reported outside of the acid motif in the C-terminal domain [27].

Increased expression of WNK proteins alters the quantitative effects that they have on distal ion transport in the kidney, and is the common molecular driver for the FHHt phenotype. Since the discovery of the WNK kinases, a number of groups have focused on the regulation of NCC and other transporters by WNK proteins including WNK1 and WNK4. Here, we review the current understanding of the molecular signalling pathways used by WNKs, which regulate ion transport in the distal nephron of the kidney.

Renal ion transporters such as NCC and NKCC2 are driven by the favourable Na^+^ gradient established by primary active transport through the Na^+^,K^+^- ATPase (Na^+^- and K^+^-dependent adenosine triphosphatase). NCC, NKCC1 and NKCC2 are the Na^+^ driven Cl^−^ importing transporters that contrast with a more recently discovered family of K^+^ driven Cl^−^ exporting transporters (KCC1–4) [28]. All these transporters are regulated by WNK kinases. In most cell types, the intracellular Cl^−^ concentration ([Cl^−^]_*i*_) is tightly regulated by influx of Cl^−^ through NCCs and an efflux of Cl^−^ via the KCCs. The [Cl^−^]_*i*_ is crucial for core physiological processes, such as transepithelial solute and water transport, volume regulation and neuronal excitability in neurons [29]. Cell shrinkage or a decrease in intracellular ([Cl^−^]_*i*_), or both, triggers the phosphorylation of NCC, NKCCs and KCCs, which leads to the activation of NCC and NKCCs and inactivation of KCCs, respectively. The converse is true when the [Cl^−^]_*i*_ is high or when the cell swells, leading to a dephosphorylation of these two sets of transporters and inactivating NCCs, and activating KCCs. Thus, the same signals achieve a tight coordination of Cl^−^ influx and efflux via the inverse regulation of Na^+^ and K^+^ driven Cl^−^ transport by a common Cl^−^-sensitive kinase in the form of the WNKs. The fact that this efficient phospho-regulatory mechanism is highly conserved from worms to humans shows how important it is for cell function and survival [30].

## Mechanisms of WNK activation, and their downstream targets

A combination of molecular genetics [14, 31, 32], physiology [19, 20, 33, 34] and biochemistry [35] has shown that the components of the signalling complex downstream of WNK kinases are serine–threonine protein kinases that share high sequence homology. The targets are SPAK (STE20/SPS1-related proline–alanine-rich protein kinase) and OSR1 (or OXSR1, oxidative stress responsive 1) that are closely related members of the STE-20 branch of the kinome (Fig. 2). The kinase domains of SPAK and OSR1 show around 89% homology, whereas at the whole protein level, the degree of homology is approximately 67%. A major difference between SPAK and OSR1 is the presence of a proline- and alanine-rich region (PAPA box) in the N-terminal domain of SPAK which is upstream of the catalytic domain [36]. There are three different isoforms of SPAK with the full-length isoform (FL-SPAK) being expressed ubiquitously with higher expression in the brain, heart, and testis [37, 38]. SPAK2, the second isoform, lacks the N-terminal PAPA box and a part of the kinase domain, and is also expressed ubiquitously. Kidney-specific SPAK (KS-SPAK) is the third isoform which is expressed mainly in the kidney, as the name suggests. Immunofluorescence studies showed that the FL-SPAK co-localized with NCC at the DCT, whereas SPAK2 and KS-SPAK are more abundant in the TAL, the site of NKCC2 expression [39].

**Fig. 3:**
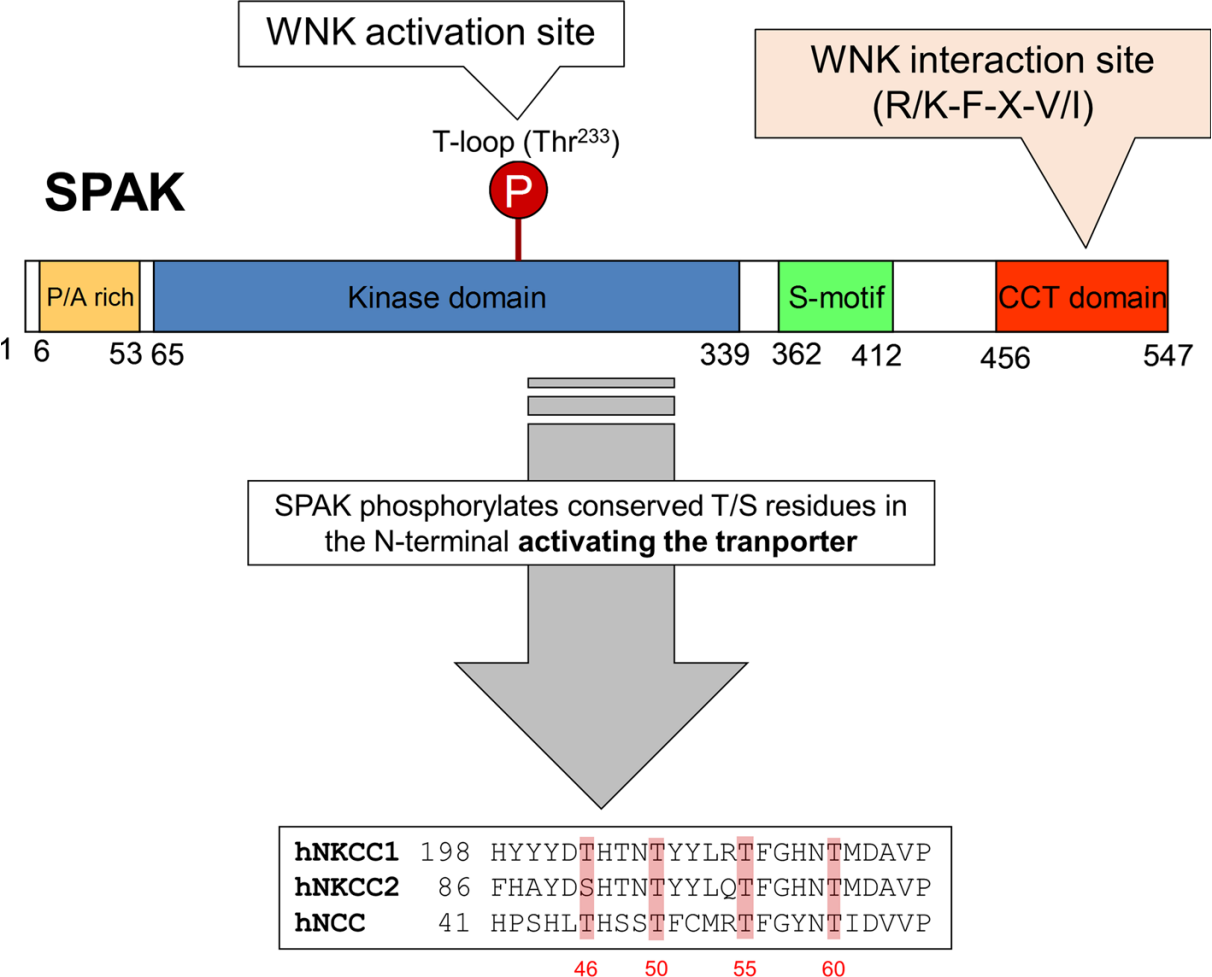
The domain structure of SPAK and the phosphorylation target sites on NCC, NKCC1 and NKCC2. OSR1 differs from SPAK in lacking the P/A rich (PAPA) domain

WNKs phosphorylate and activate SPAK and OSR1, which in turn bring about the coordinated phosphorylation of NCC and NKCC2 in the DCT and TAL of the nephron, respectively (Fig. 3). Thus, the SPAK/OSR1 kinases continue the phosphorylation cascade that connects the WNKs and the CCCs. Activation of SPAK and OSR1 takes place in an analogous manner to WNK autophosphorylation with WNKs phosphorylating a conserved Thr residue (SPAK Thr^233^, OSR1 Thr^185^; Fig. 3) within the SPAK/OSR1 catalytic T-loop motif [19]. The Ser^383^ is also phosphorylated in SPAK although its functional consequences are unclear [40]. Furthermore, in vitro assays have shown that mouse protein-25 (MO25), which functions as a scaffold, interacts with both SPAK and OSR1, and enhances their catalytic activities [41]. In addition to the catalytic domain, the SPAK/OSR1 have a conserved C-terminal domain (CCT) which is important for docking with the RFXV/I peptide motif in the N-terminus of CCCs such as NCC and NKCCs [9, 42] (Fig. 3). The CCT domain also binds to the RFXV/I peptide motif in WNK kinases bringing them into close proximity with SPAK and OSR1 to activate them. This interaction between the CCT docking domain and WNK kinases plays a major role in blood pressure regulation. OSR1 has been shown to have a highly conserved Leu residue that lies in the base of a deep hydrophobic pocket, and this forms the crucial hydrophobic contacts with the Phe residue of the RFXI motif [43]. Consistent with this in a mouse model carrying a single point mutation (Leu502Ala), the interaction between the docking domain of SPAK and the RFXV motifs on its WNK activators or ion cotransporters is abolished. The mouse also showed reduced phosphorylation and levels of NCC/NKCC2, resulting in a ∼20 mmHg reduction in BP [44].

The realization that both the kinase and the CCT domains are crucial for SPAK/OSR1 function suggested that they could serve as a potential drug targets to screen for novel antihypertensive compounds. The potential for WNK/SPAK/OSR1 to be a druggable signalling pathway is discussed below (“WNK/SPAK/OSR as a druggable signalling pathway”).

## SPAK kinase is crucial for the phosphorylation and activity of NCC

The N-terminal tail of NCC has the minimum SPAK binding motif, RFXV/I which has a docking interaction with the C-terminal domains of SPAK/OSR1, which in turn phosphorylates NCC on three highly conserved residues, Thr^46^, Thr^55^, and Thr^60^ (human amino acid numbering; Fig. 3). It has been reported that human NCC can also be phosphorylated on its Ser^91^ residue during Cl^−^ depletion by an as yet unidentified kinase [45]. Phosphorylation of Ser^71^ (Ser^73^ in humans) is also altered in SPAK-deficient mice, suggesting that the kinase is essential for the phosphorylation of this site [46]. Although both SPAK/OSR1 kinases are able to phosphorylate NCC in vitro, SPAK is essential for the NCC phosphorylation and activation in vivo.

A number of mouse models have been generated (Table 2) to characterize the SPAK kinase function: (1) a global knockout which inactivates the full-length and truncated forms of SPAK [39, 44, 46]; (2) a kinase ablating knock-in mutant (SPAK T^243A/243A^) where a missense mutation in the T-loop of SPAK prevents its activation by WNKs, and (3) a knock-in mutant (SPAK^L502A/L502A^) which is SPAK CCT domain defective and leading to markedly reduced SPAK activity and phosphorylation of CCCs such as NCC and NKCC2 [44]. In all the models, NCC phosphorylation is markedly reduced, and both the SPAK^T243A/T243A^ and SPAK^L502A/L502A^ knock-in mutants display features of Gitelman syndrome.

**Table 2:** Mouse models in which SPAK-OSR1 have been genetically modified

Gene	Genetic modification	Effect on blood pressure	Expression and activity of NCC	Phenotype	References
*SPAK*	*SPAK* ^−*/*−^	↓ with a Na^+^ depleted diet	↓↓	Hypokalemia with K^+^ depleted diet	[39]
	*SPAK* ^−*/*−^	ND	↓↓	Vasopressin induced NCC phosphorylation No NKCC2 phosphorylation	[125]
	*SPAK* ^−*/*−^	ND	ND	Decreased NKCC2 mediated Na^+^ reabsorption	[126]
	*SPAK* ^−*/*−^	↓	↓↓	Gitelman syndrome	[46]
	*SPAK* ^−*/*−^	ND	ND	Na absorption in the TAL blunted, vasopressin stimulation of NKCC2 intact	[127]
	*SPAK* ^*T243A/T243A*^	↓	↓↓	Gitelman syndrome	[38]
	SPAK^L502A/L502A^	↓	↓↓	Gitelman syndrome	[44]
*OSR1*	*OSR1* ^−*/*−^	NA	NA	Embryonically lethal	[48]
	Kidney-specific inactivation (*KSP*-*OSR1* ^−*/*−^)	Normal	↑↑	Bartter syndrome	[47]
	*OSR1* ^+*/*−^	↓	↑↑	Bartter syndrome	[47]
*SPAK/OSR1*	*SPAK* ^−/−^/*OSR1* ^flox/flox^/Pax8-rtTA^+^/Cre^+^ double knockout (DKO)	↓	↓↓	pNKCC2 levels still high Compensatory changes in NKCC2 and NCC	[49]

↑ indicates increase, ↓ indicates decrease and number of up or down arrows the size of the effect

*NA* not applicable, *ND* not determined

Genetic inactivation of OSR1 causes embryonic lethality due to defective angiogenesis and cardiovascular development [47, 48] (Table 2). The targeted inactivation of OSR1 in the distal nephron results in hypokalemia and a mild volume depletion due to the reduced expression, phosphorylation and activity of NKCC2, clinical symptoms similar to Bartter syndrome [47]. These mice show an increased NCC and phosphoNCC expression which probably compensates for the decreased NKCC2 activity. The expression and phosphorylation of NKCC2 was reduced in both SPAK^T243A/T243A^ and SPAK^L502A/L502A^ knock-in mutants, but there was an increase in the SPAK knockout mice. This difference might be due to the inhibitory effect of shorter SPAK isoforms on NKCC2, which are present in the knock-in models but not in the knockout mice. These results show that SPAK activates NCC, and that OSR1 cannot fully compensate for its absence. A recent double knockout mouse (lacking both SPAK and OSR1 activity) supports this, but also showed that there was substantial phoshoNKCC2 still present in the medulla suggesting another unrecognised kinase is important for NKCC2 phosphorylation [49].

## SPAK as a blood pressure risk allele for essential hypertension

Essential hypertension (EH) unlike the rare single gene Mendelian forms such as FHHt is caused by the effects of tens of genes whose impact is modified by gene–gene interactions and epigenesis [50, 51]. In fact, the genes coding for proteins in the WNK signalling cascade do not feature in the genetic architecture of EH. The gene encoding SPAK, *STK39*, is a singular exception. It covers 300 kb of chromosome 2 and was first identified as a hypertension susceptibility locus in a Genome-Wide association study of the Pennsylvania Amish [52]. It was replicated in several other Amish and non-Amish Caucasian cohorts. The blood pressure effect size of the strongest *STK39* alleles was up to 3 mmHg in the Amish, but smaller and less consistent across the other groups. Some of the alleles were also relatively frequent in these populations at >9%. However, the association has not been replicated in other studies notably those using a black American cohort [53] and in the Chinese Han. Although one study in Han Chinese found association in obese not in non-obese children [54], suggesting the allele is actually an obesity risk factor. A more recent meta-analysis of almost 22,000 hypertensives has confirmed the association in Europeans and East Asians but not Black American hypertensives [55]. So, it remains unclear whether the association of BP with *STK39* alleles is a false positive one or is population specific. The latter is suggested by a study in Northeastern Chinese Han people indicating that the association in the Han is regionally distinct and involve the interplay of several *STK39* alleles (rs6749447, rs35929607 and rs3754777) [56].

While the influence of *STK39* genetic variation on blood pressure is not clear, genetic variation in its phosphorylation target, NCC, is important. Over 100 mutations in the SCL21A3 gene, which encodes for NCC, have been documented in patients with Gitelman syndrome. Amongst the missense mutations within the coding region one is highly relevant to WNK signalling: T60M (homologous to mouse T^58^). It inactivates one of the key Thr residues for NCC activation and is a frequent mutation in South Asia [57] (Fig. 3). Mice homozygous for this mutation recapitulate the Gitelman phenotype very closely [58].

## WNKs as upstream regulators of SPAK in NCC activation

WNK1, WNK3 and WNK4 are expressed in the kidney, and the *WNK1* gene produces two isoforms, a long isoform called the L-WNK1 and a shorter, kidney-specific WNK1 (KS-WNK1). The L-WNK1 contains the entire kinase domain and is expressed ubiquitously, whereas the KS-WNK1 is devoid of the kinase activity, and is expressed only in the distal nephron. Initial studies on the effect of L-WNK1 on NCC in in vitro systems such as cell lines or *Xenopus* oocytes showed that this form of WNK1 had no effect on NCC expression or activity, but it abolished the inhibitory effect of WNK4 on NCC [59, 60]. However, L-WNK1 activated SPAK by phosphorylation, which indicated that it could activate NCC in a SPAK-dependent manner [45, 61]. The relevance of these pathways in vivo could not be tested because the L-WNK1 knockout models were embryonically lethal with developmental defects in the cardiovascular system, similar to those observed in OSR1-deficient embryos (Table 2) [48, 62, 63]. However, a mouse model with human *WNK1* mutations (large deletions of the first intron of the *WNK1* gene) fully recapitulated the FHHt phenotype showing an increase in L-WNK1 specifically in the DCT and CNT, with no changes in KS-WNK1 expression. Increased NCC expression and phosphorylation was also noted, and these WNK1^+/FHHt^ mice displayed an increased level of SPAK phosphorylation in the DCT, and more abundant SPAK levels at the apical membrane of the DCT when compared with wild-type mice. The FHHt phenotype was maintained in these mice even in the absence of WNK4. Thus, this study indicated a L-WNK1/SPAK pathway for NCC activation [24, 25].

WNK3 also activates NCC by a kinase- and SPAK-dependent mechanism, similar to L-WNK1 [64, 65]. WNK3 does not activate NCC in the absence of its kinase activity, and interestingly, the kinase dead WNK3 mutant is a potent inhibitor of the cotransporter. This shows that in the absence of activation, WNKs can have an opposite effect (or dominant-negative effect) on their target protein [65, 66]. WNK3 knockout mice have a very mild phenotype, and show a slight decrease in blood pressure during salt depletion. Their kidneys have an increased expression of L-WNK1, where it probably compensates for the absence of WNK3, and thus maintains NCC phosphorylation [67].

The effect of WNK4 on NCC is paradoxical, with both in vitro and in vivo studies showing that WNK4 can behave as an inhibitor as well as an activator of NCC [68]. Although these discrepancies in the literature now have to be viewed in the knowledge that [Cl^−^] was a potential unrecognised confounder in these reports (see “Intracellular Cl^−^ modulates activity of WNK kinases”). Most in vitro studies have shown that WNK4 inhibits NCC activity by abolishing the effect of WNK1 or WNK3 on NCC [69]. In vitro studies involving *Xenopus* oocytes showed that angiotensin II (AngII) signalling increased NCC activity by abolishing the inhibition by WNK4 of the cotransporter, and this effect required AngII, its receptor AT1R, and WNK4, and was prevented by the AT1R antagonist losartan. The effect of AngII on NCC was dependent on SPAK kinase because a dominant-negative SPAK or the removal of the SPAK binding motif in NCC prevented activation of NCC by AngII signalling, and this was also reported in the mpkDCT cell expression system [70]. In vivo studies in mice have shown that WNK4 is essential for basal phosphorylation and activation of NCC through its interaction with SPAK. WNK4 inactivation resulted in a significant reduction in NCC expression and activity, and this is associated with hypokalemia and metabolic alkalosis. The absence of WNK4 also abolished the stimulatory effect of AngII on phosphorylation of SPAK and NCC [71]. The converse of this, overexpression of WNK4 in a transgenic mouse model, was consistent in producing a Gitelman-like phenotype [71].

## Intracellular Cl^−^ modulates activity of WNK kinases

**Fig. 4:**
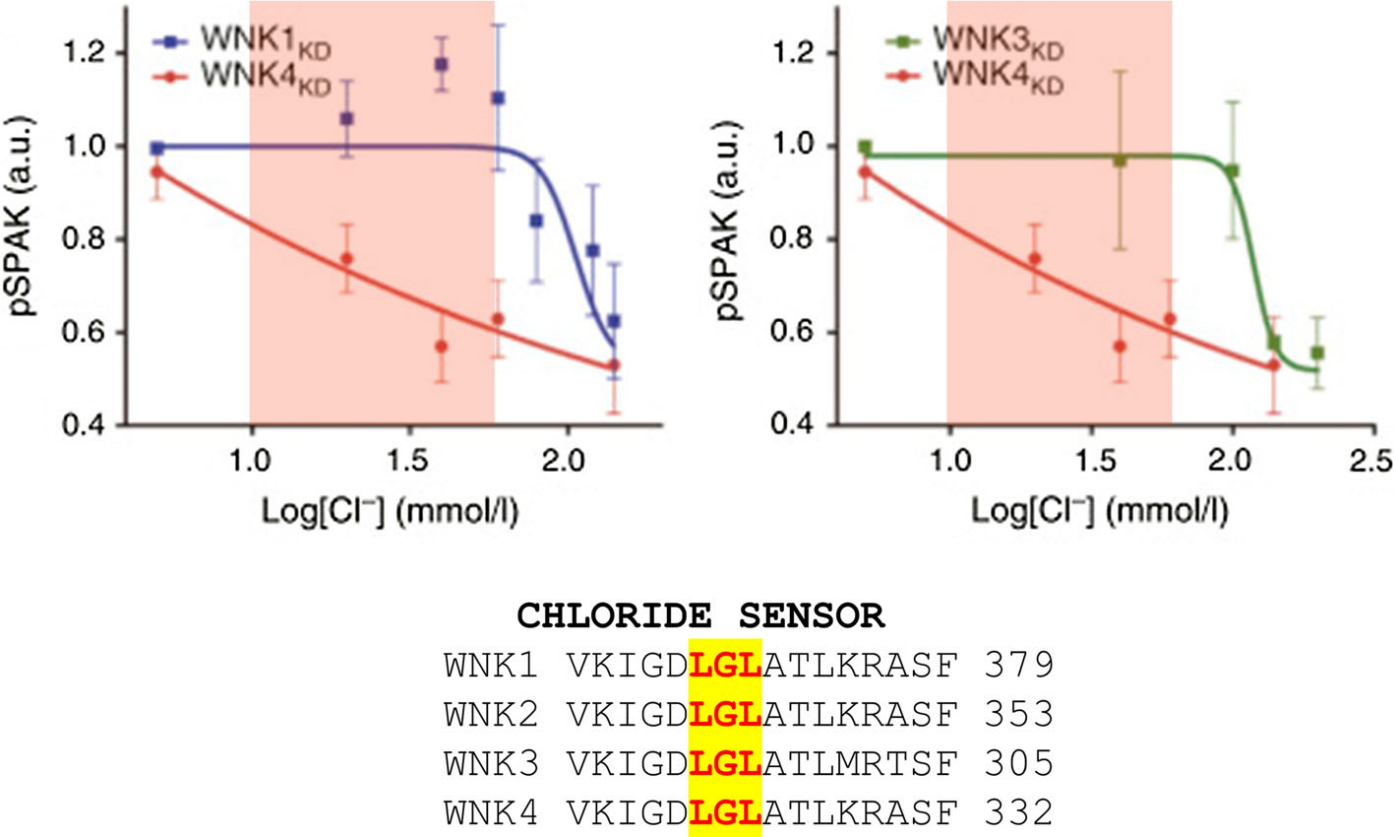
Shows the relation between the phosphorylation state of SPAK in vitro and the concentration of Cl^−^, [Cl^−^]. The latter is sensed through the chloride sensor motif on the WNKs that directly inhibits WNK kinase activity. Over the physiological range (highlighted in *pink*) WNK4 shows the greatest sensitivity to [Cl^−^]. KD here means kinase domain From reference [66] with permission

As discussed in “WNK kinases” in this review, crystallographic studies of WNK1 in its inactive state, and in the presence of Cl^−^ revealed that the anion binds directly to the catalytic domain, which could be the basis for the unique positioning of the catalytic lysine residue. This work by Piala et al. [7] showed that WNK1 fragments have a putative chloride-binding pocket formed by Leu^369^ and Leu^371^ in the DLG motif (Fig. 4), and the binding of Cl^−^ prevents WNK1 autophosphorylation. Therefore, the higher the intracellular [Cl^−^], the lower the level of autophosphorylation and hence reciprocal activation of WNK1. The effect of WNK4 on NCC within whole cells is also modulated by intracellular [Cl^−^]. A recent study in *Xenopus* oocytes has shown that WNK4 has an inhibitory effect on NCC in normal solutions, whereas when exposed to low [Cl^−^]/hypotonic conditions, it activates the NCC and thus promotes Cl^−^ efflux and a decrease in intracellular [Cl^−^] [68]. Mutating Leu^322^ (Leu^369^ in L-WNK1) resulted in the constitutive phosphorylation and activation of WNK4, and thus stimulation of NCC by WNK4 in control conditions. Thus, intracellular [Cl^−^] modulates the inhibitory versus activating effect of WNKs on NCC [68] and the discrepancies previously reported in vitro (see “WNKs as upstream regulators of SPAK in NCC activation”) probably reflect unrecognised differences in intracellular [Cl^−^]. Work by Terker et al. [72] has further shown that although the Cl^−^ binding pocket is conserved by all the WNK kinases, it is WNK4 that has the highest sensitivity to [Cl^−^]_*i*_. This is especially striking over the physiological range thought to exist within a DCT cell (10–60 mM) (Fig. 4) [73].

## Extracellular K^+^ as the ultimate regulator of WNK/SPAK/NCC pathway

**Fig. 5:**
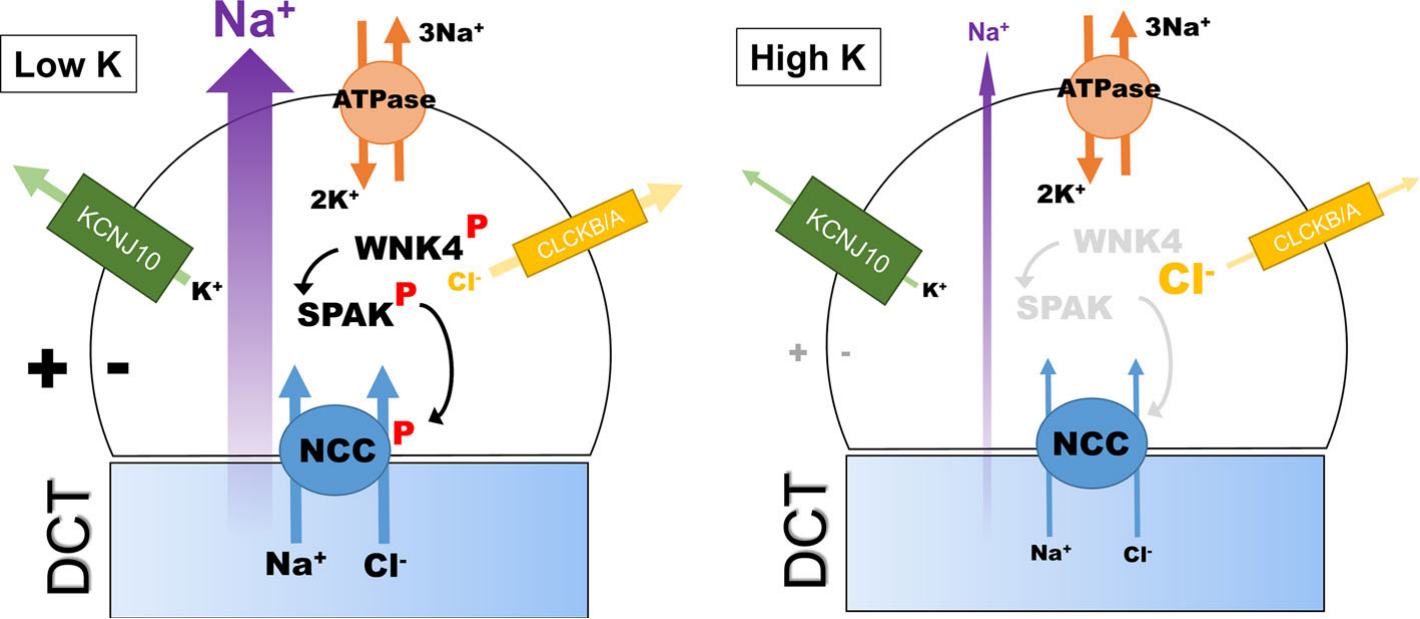
Diagram showing the hypothetical model for regulation of NCC phosphorylation in the DCT cell, and hence the level of transcellular NaCl flux. Activity in the WNK/SPAK/NCC pathway is directly regulated through the extracellular [K^+^], causing parallel changes to intracellular [Cl^−^] and WNK4 kinase activity

Recent work from the Ellison group has provided compelling evidence that the activity of the WNK/SPAK signalling pathway in the DCT is regulated by the plasma K^+^ or more precisely the concentration of K^+^ in the peritubular fluid [72]. It does this through its effects on the membrane potential of DCT cells (Fig. 5). Using HEK cells expressing NCC as a model for the DCT cell, they showed that levels of phosphoNCC and phosphoSPAK were directly affected by the extracellular K^+^ with a low K^+^ increasing the levels of both proteins. This effect of K^+^ was inhibited by both Rb^+^ and Ba^2+^ (to block K channels). Since Kir 4.1(KCNJ10; Fig. 5) is the predominant K channel in the DCT, they further showed that if mutants of this channel (which cause a Gitelman-like syndrome in humans) were expressed in the HEK cells it both depolarized them and reduced phosphoNCC levels.

A previous model for the DCT that incorporates a basolateral KCl cotransporter and a CIC channel predicted that depolarization would reduce intracellular [Cl^−^] [74], and they were able to demonstrate this in their HEK model using a chloride-sensitive fluorescent dye. Discovery of a chloride sensor in WNKs (see “WNK kinases” and “Intracellular Cl^−^ modulates activity of WNK kinases”) provides an elegant explanation for coupling of extracellular [K^+^] to NCC function through alteration in intracellular [Cl^−^]. The Ellison group tested this directly by expressing WNKs with mutated chloride sensor motifs to render them insensitive to intracellular [Cl^−^] [72]. The HEK cells no longer responded to changes in extracellular [K^+^] and showed increased levels of phosphoWNK consistent with unrestricted WNK autophosphorylation. They also showed that this derepressive effect of chloride sensor mutation was much larger for WNK4 than other WNKs [72]. This is in keeping with the in vitro sensitivity of the WNK4 to [Cl^−^] (Fig. 4) and probably explains why WNK4 has become the most important WNK for DCT function.

The model proposed by Ellison has been partly verified using a more physiological approach using perfused mouse kidney and ex vivo slices [73]. Once again low extracellular [K^+^] causes rapid phosphorylation of NCC through WNK/SPAK/OSR1 pathway activation. Using microperfused DCT tubules, the authors were also able to show that membrane potential changes at the luminal surface were most important. However, in contrast to Ellison they found that the dephosphorylation that occurs with high extracellular [K^+^] was not dependent on Cl^−^. This suggests that the dephosphorylation is regulated by so far undisclosed signalling molecules that are not part of WNK/SPAK signalling.

## CUL3–KLHL3 as upstream regulators of WNK kinases

**Fig. 6:**
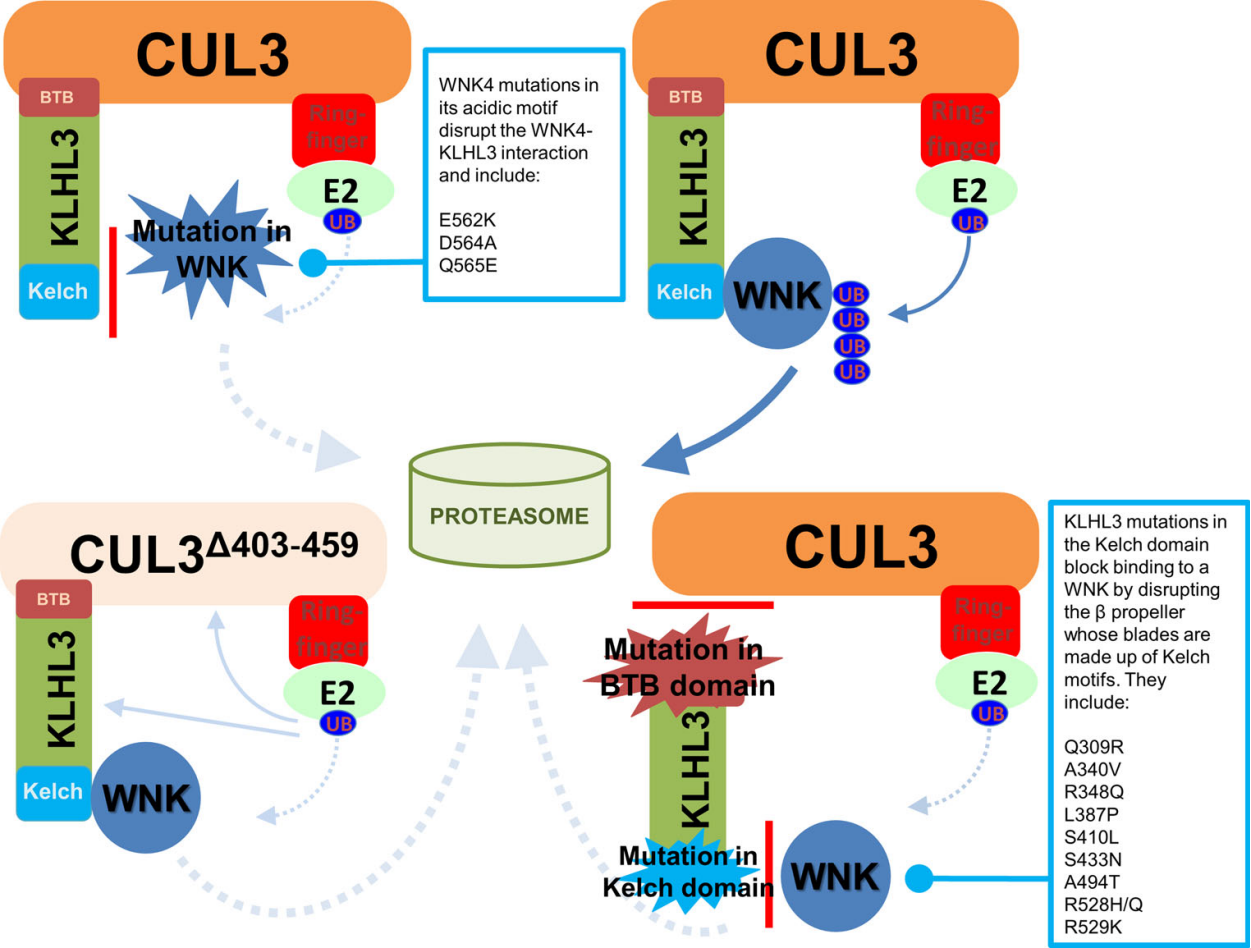
Cartoon to show the effect of different mutations in WNKs, KLHL3 or CUL3 on the docking of WNKs with the Kelch-like-3/Cullin-3-RING type E3 ubiquitin ligase complex. The situation for the wild-type is shown at *top right*. All the mutations ultimately block WNK ubiquitination, and hence its degradation by the proteasome

While mutations in *WNK1* and *WNK4* are known to cause FHHt, only about 13% of the affected pedigrees show mutations in these two genes [75]. As discussed in the introduction, recent studies have reported that mutations in *CUL3* and *KLHL3* also cause FHHt [15, 18]. CUL3 belongs to a protein family that consists of seven Cullins (Cul1, −2, −3, −4a, −4b, 5, and −7), which are all involved in the degradation of intracellular proteins by forming ubiquitin E3 ligases [76, 77]. These so-called Cullin-RING ligases share a common architecture, in which the Cullins act as the scaffold for nucleation of other E3 ligase subunits (Fig. 6). Ubiquitin E3 ligases are the final enzymes in an enzymatic cascade that leads to the ubiquitination of target proteins. Prior to ligation by E3s, ubiquitin is activated by an E1 activating enzyme and transferred to E2 conjugating enzymes [78]. The E3s are the most critical step, as they provide substrate specificity and are also often subject to regulation. The Cullins themselves are 80–100 kDa in size and structurally consist of an elongated N-terminal domain and a globular C-terminal domain. The very N-terminus of the Cullin binds to substrate adaptor and receptor proteins to recruit the substrate for ubiquitination. The E3 ligase activity resides in the C-terminus and is mediated by a small RING finger protein, either Rbx1 or Rbx2, which stably interacts with the Cullin C-terminus and recruits ubiquitin-charged E2 enzymes to transfer ubiquitin onto the substrate protein [79] (Fig. 6). Because of their modular setup, the Cullin-RING E3s are the largest class of ubiquitin E3 ligases in mammals.

Most of the seven Cullin proteins interact with their own unique set of substrate adaptor and receptor subunits. In the case of Cullin3, these are the so-called broad complex/tramtrack/bric-a-brac (BTB) proteins [76], which are characterised by BTB domains that mediate the interaction with CUL3. One subclass of BTB proteins are the Kelch-like (KLHL) proteins, a family of more than 40 Cullin3 substrate adaptors/receptors, which includes KLHL3. Kelch proteins connect the substrates to the Cullin3-RING ubiquitin ligase through their Kelch domains (Fig. 6) [80]. Kelch domains form beta propellers and X-ray crystal structures of the KLHL3 Kelch domains bound to WNK peptide revealed multiple sites of interaction, which involve residues that are mutated in FHHt patients in either WNK4 or KLHL3 [16, 17]. Indeed, mutant WNK4 proteins carrying mutations within the so-called acidic motif cannot be immunoprecipitated with KLHL3 in vitro (Ref. [17]).

Mutations in both CUL3 and KLHL3 are predicted to impair the degradation of WNK kinases. The majority of FHHt-linked KLHL3 mutations either disrupt binding to CUL3 or WNKs (see Fig. 6) to prevent the formation of an active E3 ligase. FHHt-causing mutations in KLHL3 directly block their interaction with WNK4, which reduces its ubiquitination and levels in both mammalian cells lines and kidney lysates from a mouse model expressing a typical acid motif FHHt mutation (WNK4^D561A^ [81]). This provides an elegant molecular explanation for how these mutations stabilise WNKs by preventing their degradation by ubiquitination and removal by the proteasome [17]. The findings that these mutations also cause FHHt strongly support the idea that both WNK1 and WNK4 activate NCC [15, 18, 82]. Further, knock-in mice with FHHt-like mutations in KLHL3 [R528H] have been reported to have an increased abundance of WNK1 and WNK4 proteins and increased NCC activity. In this model, Arg528 that makes critical interactions with the WNK4 degron motif is mutated, and the mice displayed a marked increase in blood pressure [83]. This presumably caused activation of the WNK/SPAK/OSR1 kinase cascade and hypertension through excess activity of the WNK/SPAK/NCC pathway.

Mutations in both CUL3 and KLHL3 are predicted to impair the degradation of WNK kinases. In fact, most FHHt-linked KLHL3 mutations either disrupt binding to CUL3, which stops assembly of the E3 complex, or to WNKs, which prevents WNK recruitment to the E3 (Fig. 6). Both mechanisms ultimately result in a loss of WNK ubiquitylation and degradation [17]. The findings that these mutations also cause FHHt strongly support the idea that both WNK1 and WNK4 activate NCC [15, 19, 73]. Further, knock-in mice with FHHt-like mutations in KLHL3 [R528H] have been reported to have an increased abundance of WNK4 proteins and increased NCC activity. In this model, Arg528 that makes critical interactions with the WNK4 degron motif is mutated, and the mice displayed a marked increase in blood pressure and increased abundance of WNK1 and WNK4 isoforms [74]. Similarly, transgenic heterozygous mice expressing a version of WNK4 that carried a familial FHHt mutation in the KLHL3 binding site (D561A/+) displayed increased WNK4 levels and overexpression of WNK4 in transgenic mice leads to FHHt-like phenotypes [81]. This stabilization of WNKs presumably caused activation of the WNK/SPAK/OSR1 kinase cascade and hypertension through excess activity of the WNK/SPAK/NCC pathway. Furthermore, it is unlikely that NCC and SPAK are directly regulated by CUL3/KLHL3, as neither protein interacts with KLHL3 [17]. This strongly suggests that the FHHt phenotype is mediated by a loss of WNK ubiquitylation and degradation, which can be caused by mutations in either the ubiquitin E3 or its WNK substrates.

Interestingly, although FHHt patients with mutations in CUL3, KLHL3 or WNK present the same clinical symptoms, those with mutations in CUL3 have a more severe phenotype, evident in terms of both an earlier age-of-onset and the degree of hypertension and electrolyte disturbance reported [15, 84, 85]. So far, all the reported FHHt CUL3 mutations show the deletion of exon 9 in the CUL3 mRNA which results in the loss of amino acid residues 403–459 in the protein. These mutations are heterozygous and autosomal dominant [15, 84, 85]. Two different mouse models of CUL3 have been reported, to date. The first is a nephron-specific deletion of CUL3 that showed the expected increase in WNK kinase levels as well the phosphorylated form of NCC [86]. However, the absence of CUL3 lead to an extended phenotype that is not typical of an FHHt phenotype. Specifically, the mice showed renal dysfunction with hypochloremic alkalosis, diabetes insipidus, and salt-sensitive hypotension. Levels of NKCC2 and AQP2 were depleted, and the loss of CUL3 leads to a widespread tubulointerstitial inflammation and fibrosis within the kidney. The second model is a knock-in mouse model for the exon 9 deletion of CUL3 which exhibited a typical FHHt phenotype, and the absence of homozygous knock-in mice for the exon 9 deletion suggested that this mutation is lethal in utero as a homozygote [16]. The heterozygous animals, CUL3^WT/Δ403–459^ exhibited significantly higher blood pressure, and an up-regulated WNK kinase pathway similar to the WNK4 mouse models for FHHt (WNK4 D568E [87, 88] and KLHL3 R528H [89]). They showed elevated levels of urine electrolytes with hyperkalemia, hyperchloremia and a compensated metabolic acidosis. These mice had higher aldosterone levels which are more likely driven by the hyperkalemia. Interestingly, in addition to the increased absorption of salt through NCC in the DCT, the CUL3^WT/Δ403–459^ mice displayed a thickened aortic wall, altered aortic pulse pressure waveform and in vivo responses to pressor agents that together suggested these mice have an increased contractile state in their vasculature, Thus, the novel vascular phenotype in the CUL3^WT/Δ403–459^ mice, in addition to the hypertension due to salt retention could possibly explain why FHHt due to CUL3 mutations result in a more severe form of the disease in comparison to FHHt due to mutations in WNK1, WNK4, KLHL3.

The vascular phenotype of the CUL3^WT/Δ403–459^ mice may have been anticipated from work done on families with mutations in the nuclear hormone receptor peroxisome proliferator-activated receptor gamma gene (*PPARγ*). These patients have an intriguing phenotype that includes lipodystrophy, metabolic syndrome and severe insulin resistance, but they also have early onset and severe hypertension [90]. The molecular basis for the hypertension was studied in a transgenic mouse model [91], which showed increased RhoA and Rho-kinase (ROCK) activity in its aorta and an increased contractile state to its of vascular ex vivo. RhoA protein (the ROCK GTPase regulator) is ubiquitinated and degraded in an analogous manner to WNKs except that the adaptor/substrate protein is not KLHL3 but the related adaptor protein RhoBTB1. This suggested that contraction reflected activation of the phosphorylation cascade through ROCK that controls myosin light chain activation in vascular smooth muscle. Hence, the deletion mutation in Cullin3 would be predicted to alter the contractile state of blood vessels by reducing Cullin3-RING ligase activity causing secondary accumulation of RhoA (cf WNKs). This scenario would also suggest that the abnormal vascular phenotype in FHHt is restricted to pedigrees with *CUL3* but not *KLHL3* mutations.

The molecular consequences of the exon 9 deleted form of mutant Cullin3 have been explored by several groups. Araki et al. [92] attempted to produce knock-in mouse models expressing the exon 9 deleted protein by knocking in two CUL3 human mutations that affect splicing: G(−1)A/+ and 3T(−6)G/T(−6)G [92]. Neither of the models produced the expected CUL3^Δ403–459^ protein, presumably because of species differences in CUL3 splicing. Instead, the mutations appeared to behave as null alleles with the total CUL3WT protein being suppressed by 50 and 25%, respectively. Importantly, neither mouse showed an FHHt phenotype. The authors reported that no truncated forms of CUL3 were detected in their mice, although close inspection of the published western blots (Fig. 4A; Ref [92]) suggests there were faint bands from a lower MW form. Of note, the low abundance of the truncated CUL3^Δ403–459^ protein was similarly difficult to demonstrate in the mouse reported by Schumacher et al. [16]. Hence, it is possible that splice processing in the models produced by Araki et al. generated minor truncated forms of CUL3 that did not mimic either the Schumacher mouse or the human processing defect.

The exon 9 deleted mutant Cullin3 protein has also been overexpressed in HEK cells to study its effects. This appeared to show that it produced reduced RhoA ubiquitination and degradation suggesting a dominant-negative effect against wild-type Cullin3 protein in vivo [93]. However, the dominant-negative effect observed in HEK cells may be secondary to marked substrate adaptor protein depletion. It is also likely that in FHHt patients with CUL3^Δ403–459^ there are very low tissue levels of the exon 9 deleted Cullin3 [16]. Structural modelling combined with biochemical verifications has also shown that the CUL3^Δ403–459^ mutation provides the Cullin3 protein with greater flexibility, which probably arises from the exon deletion splicing together two unstructured regions by removing three alpha helices. The greater flexibility impedes its scaffolding function, and prevents the CUL3^Δ403–459^-RING ubiquitin ligase complex from directing ubiquitin towards the bound substrates, and instead leads to increased ubiquitination of itself and Kelch3. In fact, in vivo studies indicate that the CUL3^Δ403–459^ auto-ubiquitinates itself first, as only relatively low amounts of CUL3^Δ403–459^ are detectable in tissue from the mouse model, while the levels of KLHL3 are unaffected. This auto-degradation of CUL3^Δ403–459^ could be a major cause for the associated phenotype in patients.

Data from in vitro studies [16] show detectable CUL3^Δ403–459^ in cells, but this protein is unable to ubiquitinate WNK kinases, suggesting that the exon 9 deletion in CUL3 is a loss of function mutation with respect to substrate ubiquitination. Yet the mutant CUL3^Δ403–459^ protein also failed to inhibit CUL3^WT^, so it may not exert a dominant-negative effect in vitro. So how is the CUL3^Δ403–459^ mutation able to produce its effect in vivo? FHHt patients with the CUL3^Δ403–459^ mutation are heterozygotes, and therefore retain a functional copy of the gene. This means the CUL3^Δ403–459^ mutation either behaves as a dominant-negative to inhibit CUL3^WT^ or it is haplo-insufficient with respect to blood pressure regulation, with a single copy of CUL3 being unable to fulfil its physiological functions including WNK degradation. Nevertheless, the work published to date does not resolve which of these scenarios is correct and more work is needed.

Blood pressure is maintained by tightly regulated ion transporters in the epithelia of the distal nephron, which balance the influx and efflux of salt, and the activities of these transporters depend on their phosphorylation by WNK kinases, as discussed in this review. The phosphorylation of NCC/NKCC2 by WNK kinases is controlled by two complementary mechanisms: the regulation of total WNK protein levels by CUL3–KLHL3, and the level of WNK activation by phosphorylation. To date, the mechanisms that sense either the need for the phosphorylation of WNK kinases or their degradation by CUL3–KLHL3 is not known, or this would be an area for future research.

The discovery of a vascular phenotype in the CUL3^WT/Δ403–459^ mice takes the role of WNK kinases in blood pressure regulation outside of the kidney. What needs confirmation is whether this vascular phenotype is a primary phenomenon or secondary to hypertension driven by NaCl retention from NCC activation. The CUL3^WT/Δ403–459^ mice have an altered aortic pulse waveform and slowed diastolic relaxation that is consistent with stiffening of their arterial tree. An increase in the contractile state of the vasculature due to wall thickening possibly contributes to the hypertension in addition to increased salt reabsorption at the distal nephron in these mice [16]. The WNK1/WNK3/SPAK/OSR1 pathway is thought to be important for regulating vascular tone by controlling the phosphorylation state of the NKCC1 cotransporter, and hence the membrane potential of vascular smooth muscle (VSM) cells [46, 94, 95]. The role of WNK/SPAK kinases in smooth muscle contraction, and angiogenesis is discussed in detail in “WNK/SPAK signalling and vascular smooth muscle contraction” and “WNK signalling and angiogenesis” of this review.

## Non-kinase dependent effects of WNKs

The WNKs regulate expression of several channels and transporters that may directly or indirectly impact on the regulation of blood pressure, e.g. the Cystic Fibrosis Conductance Regulator (CFTR), renal outer medullary K channel (ROMK) and the epithelial Na^+^ channel (ENaC). These effects are generally kinase-independent and cause altered expression of the target proteins at the cell surface. They may also operate in tandem with kinase-dependent effects through WNK/SPAK/OSR1 signalling, although they have distinct time domains. Hence, in the case of NCC, altered expression at the cell surface occurs within 15 min of exposure to ATII [96], requires WNK4 and represents altered trafficking. In contrast, the activation of NCC by phosphorylation in the same system is only apparent after 60 min of ATII stimulation and requires SPAK reflecting WNK/SPAK signalling activation.

In terms of molecular mechanisms, WNK4 reduces NCC expression at the cell surface by diverting the forward trafficking of NCC from the trans-golgi network to lysosomes. It does this by increasing complex formation with the adaptor protein AP-3 that is part of a cargo delivery system moving proteins to the lysosome compartment [97]. This makes use of YXXφ recognition motifs present in the N and C-terminal sequence of NCC. It is unclear to what extent this mechanism is generalizable to the trafficking of other WNK targets whose cell surface expression is affected. Certainly, the interaction of WNKs with CFTR, ENaC and ROMK channels do not follow this model.

Expression of the CFTR in *Xenopus* oocytes is reduced by WNKs with WNK4 reducing channel expression at the cell surface through a kinase-independent mechanism [98]. However, the effect of WNKs on CFTR expression is dependent on context as more recent work has highlighted a kinase called spleen tyrosine kinase (SYK) as having an important negative regulatory role in modulating CFTR channel expression [99, 100]. It does this through phosphorylation of a single tyrosine residue (Y^512^) close to the common mutation site in CFTR (F^508^). There is cross-talk between SYK and WNKs with WNK4 sequestering SYK and preventing Y^512^ phosphorylation of the CFTR channel [99]. The alteration of CFTR expression can impact BP and both cystic fibrosis patients and mice with reduced CFTR expression have lower BP [101]. It has been speculated that the prevalence of CF mutations in the population reflects their protective effect against the development of hypertension. Nevertheless, the exact mechanism for the hypotensive effect of reduced CFTR expression is not clear as the CFTR is widely expressed in transporting epithelia in the gut, sweat glands, kidney and lung. Recent work in mice even suggests this may be due to reduced aortic contractility by affecting calcium mobilization [102].

The ENaC channel is expressed in the distal nephron where it regulates Na^+^ reabsorption giving the channel a pivotal role in salt homeostasis and the long-term control of BP. Expression and activity of ENaC is regulated by the hormones vasopressin (ADH) and aldosterone. Vasopressin recycles channels to the cell surface from a recycling pool [103] while aldosterone drives the synthesis of new ENaC channels and a kinase, serum-glucocorticoid regulated kinase 1 (SGK1), that stabilises expression of ENaC at the cell surface [104–106]. The ENaC channels are normally cycled from the cell surface after ubiquitination by the ubiquitin E3 ligase Nedd4-2 and phosphorylation of Nedd4-2 by SGK1 reduces its interaction with ENaC [105]. The WNKs interact at several points in this process. First, WNK1 can bind and activate SGK1, although this is not a catalytic effect [107] and seems to be common property of the N-terminal of all four WNKs [108]. WNK4 also reduces ENaC expression at the cell surface. This effect is independent of Nedd4-2 so that it is able to reduce surface expression of ENaC subunits that lack the YY motifs necessary for interaction with Nedd4-2 [109]. Finally, WNK4 is itself a substrate for SGK1, although its phosphorylation at Ser1169 confers the unusual property of reversing its action against ENaC [110].

The ROMK channel is expressed only in the kidney. In the distal nephron, ROMK functions as an important secretory channel for K^+^. Since serum K tightly regulates the synthesis and release of aldosterone ROMK expression can also modulate BP. It also directly impacts Na^+^ homeostasis as Na^+^ reabsorption in the thick ascending limb of the loop of Henle is limited by local K^+^ recycling through ROMK. The surface expression of ROMK is again suppressed by WNKs, but the molecular details are distinct from their effects on ENaC trafficking. Specifically, ROMK is not internalised by WNKs (WNK1 and WNK4) through an ubiquitination pathway. Instead, accelerated internalisation of ROMK is dynamin-dependent and involves clathrin-coated pits [111, 112].

## WNK/SPAK signalling and vascular smooth muscle contraction

The role of the NKCC1 cotransporter in chloride-transporting epithelia and in the control of cell volume [113] is well established. Less well appreciated is its role in the vasculature in controlling blood vessel tone. Although, Cl^−^ entry into smooth muscle cells generally depolarises the membrane potential and contracts them [114]. NKCC1 is the only SLC12A3 sodium-chloride cotransporter expressed in the aorta, and its key role in Cl^−^ entry into vascular smooth muscle was confirmed by deletion of the transporter in the mouse. The blood pressure in *NKCC1* ^−/−^ mice was substantially lower than wild-type controls [115]. Electrolyte and aldosterone levels were unaffected in this model implying that salt and water homeostasis was intact. However, venous smooth muscle ex vivo showed less tone and a reduced vasorelaxation to bumetanide. A subsequent study has shown an effect of NKCC1 inhibition on the resistance vessels as well [116]. The components of the WNK/SPAK/OSR1 pathway are present in aortic vascular smooth muscle and NKCC1 is regulated by phosphorylation of N-terminal Ser/Thr residues homologous to those present in the N-terminus of NCC and NKCC2. Confirmation of the pathway was provided by a SPAK knockout mouse that had low blood pressure, reduced aortic phosphoNKCC1, and reduced aortic responses to phenylephrine and bumetanide. The level of phosphoNKCC1 in the aorta responds directly to dietary levels of salt in the mouse with a low-salt diet increasing phosphoNKCC1 [95]. This effect of dietary salt is mimicked by AngII and blocked by the ATIR receptor antagonist valsartan. Hence the WNK/SPAK/NKCC1 cascade in vascular smooth muscle appears to be regulated physiologically by the renin-angiotensin system.

There is some uncertainty over which various WNK isoforms is most important in the vessel wall. *WNK1* deletion (*WNK1* ^−/−^) is lethal in utero, but mice with haploinsufficiency for *WNK1* (*WNK1* ^+/−^) have reduced pressor responses to phenylephrine and reduced contraction in vitro to α-adrenergic contraction. Isolated vessels also showed reduced myogenic responses to load [94]. In contrast, the WNK/SPAK/NKCC1 cascade was not activated by ATII or dietary salt restriction in a WNK3 knockout mouse. This mouse also showed reduced pressor responses to infused ATII [95]. This suggests that both WNK isoforms have a role in the vessel wall with WNK1 perhaps being more important for catecholaminergic tone and WNK3 for regulation by the renin–angiotensin system.

## WNK signalling and angiogenesis

Mice with homozygous deletion of the *WNK1* gene (*WNK1* ^−/−^) die in utero before embryonic day 13 [63]. The development of the heart is abnormal and both sprouting and remodelling angiogenesis is impaired in WNK1 null embryos. By crossing Tie2-Cre with floxed *WNK1* mice, it has been shown that it is the endothelial-specific loss of WNK1 that causes the angiogenesis defects [62]. The resulting mice are striking for showing ectopic expression of venous markers in arteries and arterial markers in veins. This suggests that WNK1 is acting beyond the differentiation switch that determines whether a nascent vessel follows an arterial or venous fate. The arterial differentiation switch is dependent on the activation of VEGF/Notch signalling and the existence of cross-talk between VEGF and WNK signalling has been suggested by recent work in Zebrafish [117]. In this model vertebrate species WNK signalling is again important for the formation of the vessels of the head and trunk. Of note, knock-down of WNK1 with morpholino antisense oligos produced similar defects in Zebrafish angiogenesis to knock-down of the VEGF2 receptor itself. The VEGFR2 is the major receptor mediating VEGF effects in the vasculature and its Tyrosine Kinase (TK) function activates downstream kinases including phosphoinositide-dependent protein kinase (PI3 kinase). PI3 kinase in turn activates another kinase, Akt/PKB, which is an important kinase in mediating the metabolic and mitogenic effects of insulin. There is a clear biochemical opportunity for cross-talk between VEGF and WNK1 signalling, since the N-terminal of WNK1 has a phosphorylation consensus sequence for Akt/PKB1, and IGF-1 stimulates phosphorylation of the conserved Thr^60^ residue in this sequence in HEK cells [118]. This event seems to negatively regulate growth, since blockade of WNK1 phosphorylation increased the effect of insulin on preadipocyte cell division [119]. The ability of WNK1 mRNA to rescue the effects of VEGF2R knock-down in Zebrafish also relies on an intact Akt/PKB1 sequence to allow Thr^60^ phosphorylation. Akt/PKB1 activation of WNK1 in the kidney is also seen in db/db mice suggesting it has a role in the hypertension seen in the metabolic syndrome [120].

## VEGF/WNK signalling and hypertension

Could the interplay of VEGFR2 and WNK1 signalling pathways have a role in regulating blood pressure outside of the embryonic vasculature? The widespread use of inhibitors of VEGF in oncotherapy, both in the form of monoclonal antibodies to block VEGF receptors or small molecule inhibitors of VEGF TK receptor signalling has highlighted a high frequency of hypertension as a side effect [121]. In trials, the majority of patients actually develop hypertension especially those involving potent VEGF TK inhibitors such as axitinib [122]. The severity of the hypertension parallels the level of functional VEGF inhibition and is reversed when the inhibitor is stopped. Hence, an off-target effect seems unlikely and the degree of hypertension may even be a biomarker of a favourable treatment response. Nevertheless, the molecular mechanisms behind this pressor effect are still unclear. Endothelial dysfunction, vascular stiffening or remodelling and vascular rarefaction (a reduction in the density of microvessels) have all been suggested [121]. The hypothesis of rarefaction is perhaps the most suggestive of a mechanism reflecting VEGF/WNK signalling cross-talk. However, this has not been explored, but is attractive as the rarefaction may be blocked by drugs targeting the WNK/SPAK/OSR1 cascade (see “WNK/SPAK/OSR as a druggable signalling pathway”).

## WNK/SPAK/OSR as a druggable signalling pathway

The conserved carboxy-terminal (CCT) domain of SPAK binds with high affinity to RFXV/I motifs that are present both in its upstream activator (WNKs) as well as its downstream substrates (Fig. 3). In fact, mutation of a single highly conserved Leu502 within the CCT domain abolishes high affinity binding to the RFXI/V motif. The critical importance of SPAK docking to its binding partners for WNK/SPAK/OSR signalling was confirmed by mutation of the canonical Leu50 to alanine [44]. Mice homozygous for this mutation show marked reduction in expression and phosphorylation of NCC and NKCC2 in the kidney. The mice were also hypotensive suggesting that blocking the CCT domain with a small molecule could provide a novel antihypertensive strategy.

To identify lead compounds that block binding to the CCT domain of SPAK, Uchida’s group developed a high-throughput assay using a fluorescent RFXV/I peptide based on the motif present in either WNK1 or WNK4 as bate for a GSK–SPAK–CCT fusion protein [89]. The bait–target interaction was assessed by fluorescent correlation spectroscopy. Using this approach, they were able to identify ten candidate molecules from a 17,000 compound library with STOCK1S-50699 (PubChem-CID 5749625) and STOCK2S-26016 (PubChem-CID 3135086) having the highest activity. These two molecules were also able to block phosphorylation of SPAK and NCC in a cell-based assay. The same group also developed a second high-throughput assay employing a novel ELISA-based assay to detect inhibition of NKCC2 phosphorylation [123]. Using an extended library (totalling almost 22,000 compounds) they identified a single lead molecule (1S-14279; PubChem CID 01676700) that in the Biacore^®^ system bound to SPAK with an affinity constant of ~10^5^ M^−1^. Further work showed that the molecule was chemically similar to another molecule detected with the assay run against another smaller targeted library. This molecule was closantel, a known anthelminthic drug. Both closantel and 1S-14279 appeared to block SPAK docking to it phosphorylation target rather than compete with ATP binding. This is important for specificity of these molecules given the structural similarity of the ATP binding site throughout the family of TK proteins.

In cell-based assays, closantel and 1S-14279 were both effective at blocking NCC phosphorylation [123]. They also acutely reduced the level of phosphoNCC and phosphoNKCC1 in vivo. However, 1S-14279 appears too toxic for chronic administration and closantel, while reducing levels of phosphoproteins very significantly over 7-day administration had no effect on blood pressure or serum or urinary electrolytes. However, another very promising lead molecule has emerged very recently in the form of a substituted imidazole WNK463 [124]. This compound is able to block all of the WNKs (WNK1, WNK2, WNK3 and WNK4) in vitro in the nM concentration range. It is also orally active and reduced BP, urinary Na^+^ output and phosphorylation levels of WNK4 in mouse kidney lysates. However, it has not been developed further as a therapeutic agent due to problems with its preclinical safety profile. Nevertheless, WNK463 will be an invaluable tool molecule and provides a clear proof of principle that targeting the WNK/SPAK/OSR1 cascade can provide effective antihypertensive drugs for clinical use.

## Conclusion

The WNKs are ancient proteins in evolutionary terms and were co-opted early on to control cell volume and intracellular chloride levels by forming signalling pathways with the related kinases, SPAK and OSR1. In transporting epithelium, including the kidney nephron itself these pathways are crucial to the regulation of ion fluxes by controlling the phosphorylation state of key membrane transporters. Hence, the WNK/SPAK/OSR1 signalling pathway directly influences blood pressure, as is clearly seen in rare monogenic blood pressure syndromes mutating single genes in the cascade. Emerging evidence suggests that WNK/SPAK/OSR1 signalling also operates outside of the kidney where it is able to regulate blood vessel tone directly. Much is still to be learned about the physiology and pathophysiology of the WNKs and their signalling roles, but targeting the WNK/SPAK/NCC pathway holds considerable promise for the development of novel antihypertensive drugs and diuretics.
